# Trends in deaths from road injuries during the COVID-19 pandemic in Japan, January to September 2020

**DOI:** 10.1186/s40621-020-00294-7

**Published:** 2020-11-30

**Authors:** Shuhei Nomura, Takayuki Kawashima, Daisuke Yoneoka, Yuta Tanoue, Akifumi Eguchi, Stuart Gilmour, Masahiro Hashizume

**Affiliations:** 1 Department of Health Policy and Management, School of Medicine, Keio University, 35 Shinanomachi, Shinjuku-ku, Tokyo, 160-8582 Japan; 2 Department of Global Health Policy, Graduate School of Medicine, The University of Tokyo, Tokyo, Japan; 3 Department of Mathematical and Computing Science, Tokyo Institute of Technology, Tokyo, Japan; 4 Graduate School of Public Health, St. Luke’s International University, Tokyo, Japan; 5 Institute for Business and Finance, Waseda University, Tokyo, Japan; 6 Department of Sustainable Health Science, Center for Preventive Medical Sciences, Chiba University, Chiba, Japan

**Keywords:** COVID-19, Japan, Excess deaths, Exiguous deaths, Road injuries

## Abstract

**Background:**

In Japan, the latest estimates of excess all-cause deaths through January to July 2020 showed that the overall (direct and indirect) mortality burden from the Coronavirus Disease 2019 (COVID-19) in Japan was relatively low compared to Europe and the United States. However, consistency between the reported number of COVID-19 deaths and excess all-cause deaths was limited across prefectures, suggesting the necessity of distinguishing the direct and indirect consequences of COVID-19 by cause-specific analysis. To examine whether deaths from road injuries decreased during the COVID-19 pandemic in Japan, consistent with a possible reduction of road transport activity connected to Japan’s state of emergency declaration, we estimated the exiguous deaths from road injuries in each week from January to September 2020 by 47 prefectures.

**Methods:**

To estimate the expected weekly number of deaths from road injuries, a quasi-Poisson regression was applied to daily traffic fatalities data obtained from Traffic Accident Research and Data Analysis, Japan. We set two thresholds, point estimate and lower bound of the two-sided 95% prediction interval, for exiguous deaths, and report the range of differences between the observed number of deaths and each of these thresholds as exiguous deaths.

**Results:**

Since January 2020, in a few weeks the observed deaths from road injuries fell below the 95% lower bound, such as April 6–12 (exiguous deaths 5–21, percent deficit 2.82–38.14), May 4–10 (8–23, 21.05–43.01), July 20–26 (12–29, 30.77–51.53), and August 3–9 (3–20, 7.32–34.41). However, those less than the 95% lower bound were also observed in weeks in the previous years.

**Conclusions:**

The number of road traffic fatalities during the COVID-19 pandemic in Japan has decreased slightly, but not significantly, in several weeks compared with the average year. This suggests that the relatively small changes in excess all-cause mortality observed in Japan during the COVID-19 pandemic could not be explained simply by an offsetting reduction in traffic deaths. Considering a variety of other indirect effects, evaluating an independent, unbiased measure of COVID-19-related mortality burden could provide insight into the design of future broad-based infectious disease counter-measures and offer lessons to other countries.

**Supplementary Information:**

The online version contains supplementary material available at 10.1186/s40621-020-00294-7.

## Background

The Coronavirus Disease 2019 (COVID-19) has become a worldwide pandemic. Globally, accurate figures on COVID-19 deaths are difficult to determine due to limited availability and quality of virus testing (Pulla 2020), and many countries perform ‘excess death’ monitoring to assess the mortality burden of COVID-19 (Vestergaard, Nielsen et al. 2020). Excess deaths monitoring estimates an increase in all-cause mortality that is higher than expected under normal circumstances.

In Japan, the latest estimates of excess all-cause deaths through January to July 2020 showed that the overall (direct and indirect) mortality burden from COVID-19 in Japan was relatively low compared to Europe and the United States (National Institute of Infectious Diseases, 2020a). From January to July 2020, the national excess all-cause deaths per 100,000 population was approximately 0.2 to 6.0 in Japan, including uncertainty; on the other hand, for example, in the United States, although a complete country-comparison is not always possible given the different estimation algorithms in each country, the excess all-cause deaths from March to September were about 85 per 100,000 population, and in Spain during the similar period, those were 87 per 100,000 population. (The Economist, 2020) However, consistency between the reported number of COVID-19 deaths and excess all-cause deaths was limited across prefectures, suggesting the necessity of distinguishing the direct and indirect consequences of COVID-19 by cause-specific analysis. In particular, if mortality due to causes other than COVID-19 decreases during the pandemic, the excess of direct deaths from COVID-19 may be offset by exiguous deaths—a decrease in mortality that is lower than would be expected under normal circumstances—in some other causes, making it difficult to interpret the mortality burden of COVID-19.

However, cause-specific excess deaths monitoring cannot be conducted in a timely manner in Japan because publication of vital statistics by cause of death is delayed, as is the case throughout the world (Leon et al., 2020). Instead, we used the traffic fatality data reported by the National Police Agency to examine whether deaths from road injuries actually decreased during the COVID-19 pandemic in Japan, consistent with a reduction of road transport activity connected to Japan’s state of emergency declaration. In fact, people’s mobility at transit stations and traffic volumes on major national highways in 2020 decreased from the average year, and it was particularly large in April and May after the state of emergency was declared on April 7, with a decrease of about 30% for each (Google, 2020, Ministry of Land, Infrastructure and Transport, 2020).

## Methods

### Estimating expected number of deaths from road injuries

We obtained daily open data from 4 January 2010 to 6 September 2020 from the Institute for Traffic Accident Research and Data Analysis (ITARDA), Japan (Institute for Traffic Accident Research and Data Analysis, 2020). ITARDA gathers daily reports on the number of deaths within 24 h of a traffic accident from the National Police Agency. Daily data was converted to weekly data to ensure enough samples for analyses; the first week was from 4 to 10 January 2010 and the last week was from 31 August to 6 September 2020, based on the epidemiological week of Japan’s National Institute of Infectious Diseases’ Infectious Diseases Weekly Report (National Institute of Infectious Diseases, 2020b).

To estimate the expected number of deaths from road injuries and the associated prediction intervals, we employed the Farrington algorithm, which computes a quasi-Poisson regression model and is commonly used to study the annual and seasonal trends of the burden of disease attributable to seasonal pandemics (Vestergaard et al., 2020). The major characteristic of Farrington algorithm is to limit the data for the estimation: the expected number of deaths at a calendar week *t* is estimated using only the data during *t* − *w* and *t* + *w* weeks of years *h* − *b* and *h* − 1, where *w* and *b* are pre-fixed values and *h* is the year of *t*. In this study, we considered *b* = 5 and *w* = 3. The limited data is referred to as a reference period. In addition, to incorporate the seasonality into the model, data that is not included in the reference period is equally divided and included in the regression model as dummy variables. Then, the regression model is given by:

1 $$ {Y}_t\sim QPoisson\left({\mu}_t,{\phi \mu}_t\right),\log \left({\mu}_t\right)=\alpha +\beta t+{\boldsymbol{f}}^T(t){\upgamma}_{f(t)} $$

where *QPoisson*(*a*, *b*) is a quasi-Poisson distribution with expectation *a* and variance *b*, *Y*_*t*_ is the number of deaths at a certain week *t*, *α* and *β* are regression parameterss, ***γ***_***f***(***t***)_ is a regression parameter vector representing the seasonality, ***f***(***t***) is a vector of dummies that equally divides the time points outside the reference period into nine periods, following the previous study (Centers for Disease Control and Prevention, 2020) and upper subscript *T* denotes the transpose of a vector. The parameters, including the regression coefficients and overdispersion parameter *ϕ*, were estimated by the quasi-likelihood approach. More details can be found in Farrington et al. (1996) and Noufaily et al. (2013) (Farrington et al., 1996, Noufaily et al., 2013).

Once the regression parameters were estimated, the expected number of deaths is predicted for the week of interest *t*_0_. The two-sided 95% prediction interval was then estimated by assuming that the data follows the negative binomial distribution as ${Y}_{t_0}\sim NB\left(\hat{Y_{t_0}},\hat{\nu_0}\right)$, where $\hat{Y_{t_0}}$ is the mean of the distribution and $\hat{\nu_0}=\hat{\frac{Y_{t_0}}{\phi -1}}$ is its dispersion parameter.

### Exiguous deaths

We set two thresholds, point estimate and lower bound of the two-sided 95% prediction interval, for exiguous deaths, and report the range of differences between the observed number of deaths and each of these thresholds as exiguous deaths. The percent deficit was defined as the number of exiguous deaths divided by the threshold.

## Results

**Fig. 1:**
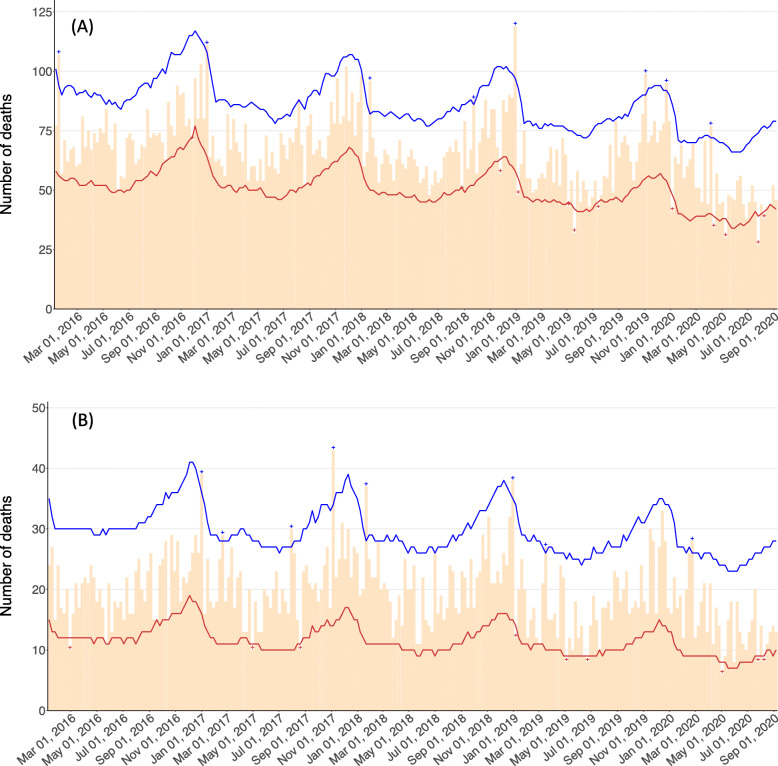
Weekly observed and 95% upper and lower bound of the expected weekly number of deaths from road injuries in Japan up to 6 September 2020 (**a**: Japan, **b**: seven prefectures where a state of emergency was first declared on 7 April 2020). Orange: observed; Blue: upper bound; Red: lower bound; cross symbols indicate weeks with the observed exceeding or falling the 95% upper or lower bounds

Since January 2020, in some weeks the observed deaths from road injuries fell below the 95% lower bound, such as 6–12 April (exiguous deaths 5–21, percent deficit 2.82–38.14), 4–10 May (8–23, 21.05–43.01), 20–26 July (12–29, 30.77–51.53), and 2–9 August (3–20, 7.32–34.41) (Fig. 1 (A)). However, those less than the 95% lower bound were also observed in weeks in the previous years, particularly in 2019, such as 29 April–5 May (2–17, 4.44–27.91), 13–19 May (10–26, 23.81–44.39), and 8–14 July (3–20, 6.67–31.40). Also, in some weeks including April 5–11, 2020, the observed deaths from road injuries exceeded the 95% upper bound. Weekly exiguous deaths since January 2020 are presented in Table 1 and from 2016 to 2019 are presented in online supplementary Table 1. Similar results were obtained in a sensitivity analysis of only the seven prefectures where a state of emergency over COVID-19 was declared in 7 April 2020 (including Tokyo) (Fig. 1 (B) and Table 2 for 2020 and online supplementary Table 2 for 2016–2019).

**Table 1:** Weekly number of observed and deficit deaths and percent deficit from road injuries in Japan from January to September 2020

Week	Week ending date	Observed deaths	Exiguous deaths	Percent deficit
1	5 January 2020	41	7–26	14.58–38.27
2	12 January 2020	64	0–0	0.00–0.00
3	19 January 2020	61	0–0	0.00–0.00
4	26 January 2020	69	0–0	0.00–0.00
5	2 February 2020	55	0–0	0.00–0.00
6	9 February 2020	60	0–0	0.00–0.00
7	16 February 2020	63	0–0	0.00–0.00
8	23 February 2020	70	0–0	0.00–0.00
9	1 March 2020	51	0–4	0.00–5.83
10	8 March 2020	51	0–5	0.00–7.53
11	15 March 2020	45	0–10	0.00–17.62
12	22 March 2020	71	0–0	0.00–0.00
13	29 March 2020	44	0–12	0.00–20.74
14	5 April 2020	77	0–0	0.00–0.00
15	12 April 2020	34	5–21	12.82–38.14
16	19 April 2020	57	0–0	0.00–0.00
17	26 April 2020	44	0–9	0.00–16.30
18	3 May 2020	45	0–8	0.00–15.02
19	10 May 2020	30	8–23	21.05–43.01
20	17 May 2020	48	0–3	0.00–5.76
21	24 May 2020	47	0–3	0.00–4.13
22	31 May 2020	46	0–4	0.00–6.85
23	07 June 2020	54	0–0	0.00–0.00
24	14 June 2020	56	0–0	0.00–0.00
25	21 June 2020	44	0–7	0.00–12.50
26	28 June 2020	38	0–14	0.00–26.08
27	5 July 2020	45	0–8	0.00–14.91
28	12 July 2020	52	0–3	0.00–4.83
29	19 July 2020	45	0–12	0.00–19.69
30	26 July 2020	27	12–29	30.77–51.53
31	2 August 2020	44	0–13	0.00–22.54
32	9 August 2020	38	3–20	7.32–34.41
33	16 August 2020	43	0–16	0.00–26.75
34	23 August 2020	44	0–16	0.00–26.26
35	30 August 2020	52	0–9	0.00–13.76
36	6 September 2020	46	0–14	0.00–22.38

Percent deficit during the COVID-19 pandemic were defined as the number of deficit deaths divided by the threshold. Weeks with observed deaths from road injuries falling the 95% lower bound were highlighted in gray

**Table 2:** Weekly number of observed and deficit deaths and percent deficit from road injuries in seven prefectures where a state of emergency was first declared on 7 April 2020, from January to September 2020

Week	Week ending date	Observed deaths	Exiguous deaths	Percent deficit
1	5 January 2020	16	0–7	0.00–27.32
2	12 January 2020	22	0–0	0.00–0.00
3	19 January 2020	17	0–1	0.00–5.53
4	26 January 2020	19	0–0	0.00–0.00
5	2 February 2020	12	0–6	0.00–31.72
6	9 February 2020	20	0–0	0.00–0.00
7	16 February 2020	26	0–0	0.00–0.00
8	23 February 2020	28	0–0	0.00–0.00
9	1 March 2020	12	0–6	0.00–30.28
10	8 March 2020	14	0–4	0.00–18.35
11	15 March 2020	18	0–0	0.00–0.00
12	22 March 2020	21	0–0	0.00–0.00
13	29 March 2020	13	0–5	0.00–24.42
14	5 April 2020	21	0–0	0.00–0.00
15	12 April 2020	11	0–6	0.00–31.99
16	19 April 2020	17	0–0	0.00–0.00
17	26 April 2020	14	0–2	0.00–9.10
18	3 May 2020	6	2–10	25.00–60.80
19	10 May 2020	9	0–7	0.00–41.66
20	17 May 2020	15	0–0	0.00–0.00
21	24 May 2020	18	0–0	0.00–0.00
22	31 May 2020	8	0–7	0.00–44.01
23	07 June 2020	18	0–0	0.00–0.00
24	14 June 2020	13	0–2	0.00–13.16
25	21 June 2020	11	0–5	0.00–27.20
26	28 June 2020	10	0–6	0.00–34.07
27	5 July 2020	13	0–3	0.00–17.30
28	12 July 2020	15	0–2	0.00–7.86
29	19 July 2020	20	0–0	0.00–0.00
30	26 July 2020	8	1–9	11.11–51.16
31	2 August 2020	14	0–4	0.00–18.16
32	9 August 2020	8	1–10	11.11–54.10
33	16 August 2020	11	0–7	0.00–38.36
34	23 August 2020	13	0–5	0.00–26.40
35	30 August 2020	14	0–4	0.00–21.60
36	6 September 2020	13	0–6	0.00–27.89

Percent deficit during the COVID-19 pandemic were defined as the number of deficit deaths divided by the threshold. Weeks with observed deaths from road injuries falling the 95% lower bound were highlighted in gray

## Discussion

Although the age-specific analysis, for which data were not available in this study, may yield different results, the number of road traffic fatalities for all ages during the COVID-19 pandemic has decreased slightly, but not significantly, in several weeks compared to the average year in Japan. Other limitations of this study include that it was not possible to analyse the circumstances under which the traffic accident occurred or to analyse data on deaths within 30 days of the accident, which can refer to cases where a patient died because of a shortage of intensive care unit beds due to COVID-19 and was not treated in time. Our findings suggest that the relatively small changes in excess all-cause mortality observed in Japan could not be explained simply by an offsetting reduction in traffic deaths. Considering a variety of other indirect effects, evaluating an independent, unbiased measure of COVID-19-related mortality burden could provide insight into the design of future broad-based infectious disease counter-measures and offer lessons to other countries (Vestergaard and Molbak, 2020).

## Supplementary Information

**Additional file 1: Table S1.** Weekly number of observed and deficit deaths and percent deficit from road injuries in Japan from January 2016 to 2019. **Table S2.** Weekly number of observed and deficit deaths and percent deficit from road injuries in seven prefectures where a state of emergency was first declared on 7 April 2020, from January 2016 to 2019.

## Data Availability

Data are openly available in the Institute for Traffic Accident Research and Data Analysis’s website at https://www.itarda.or.jp/english/report.

## Abbreviations

COVID-19: Coronavirus Disease 2019
ITARDA: Institute for Traffic Accident Research and Data Analysis
