# Supplementary material for: Trends in deaths from road injuries during the COVID-19 pandemic in Japan, January to September 2020
Source: Inj Epidemiol. 2021 Jan 18;7:66. doi: 10.1186/s40621-020-00294-7 (PMC7703507; doi:10.1186/s40621-020-00294-7)
Supplement: Supplementary file 1 — Additional file 1: Table S1. Weekly number of observed and deficit deaths and percent deficit from road injuries in Japan from January 2016 to 2019. Table S2. Weekly number of observed and deficit deaths and percent deficit from road injuries in seven prefectures where a state of emergency was first declared on 7 April 2020, from January 2016 to 2019. [file 40621_2020_294_MOESM1_ESM.docx]

**Supplementary table 1: Weekly number of observed and deficit deaths and percent deficit from road injuries in Japan from January 2016 to 2019.**

| 2016 |  |  |  |  |
| --- | --- | --- | --- | --- |
| Week | Week ending date | Observed deaths | Exiguous deaths | Percent deficit |
| 1 | 10 January 2016 | 77 | 0–2 | 0.00–1.96 |
| 2 | 17 January 2016 | 107 | 0–0 | 0.00–0.00 |
| 3 | 24 January 2016 | 57 | 0–15 | 0.00–20.48 |
| 4 | 31 January 2016 | 71 | 0–2 | 0.00–2.60 |
| 5 | 7 February 2016 | 62 | 0–12 | 0.00–15.63 |
| 6 | 14 February 2016 | 67 | 0–7 | 0.00–9.15 |
| 7 | 21 February 2016 | 68 | 0–6 | 0.00–6.99 |
| 8 | 28 February 2016 | 60 | 0–12 | 0.00–16.20 |
| 9 | 6 March 2016 | 61 | 0–11 | 0.00–14.14 |
| 10 | 13 March 2016 | 81 | 0–0 | 0.00–0.00 |
| 11 | 20 March 2016 | 68 | 0–4 | 0.00–4.35 |
| 12 | 27 March 2016 | 75 | 0–0 | 0.00–0.00 |
| 13 | 3 April 2016 | 67 | 0–4 | 0.00–5.40 |
| 14 | 10 April 2016 | 74 | 0–0 | 0.00–0.00 |
| 15 | 17 April 2016 | 70 | 0–1 | 0.00–0.46 |
| 16 | 24 April 2016 | 76 | 0–0 | 0.00–0.00 |
| 17 | 1 May 2016 | 74 | 0–0 | 0.00–0.00 |
| 18 | 8 May 2016 | 84 | 0–0 | 0.00–0.00 |
| 19 | 15 May 2016 | 69 | 0–0 | 0.00–0.00 |
| 20 | 22 May 2016 | 67 | 0–0 | 0.00–0.00 |
| 21 | 29 May 2016 | 78 | 0–0 | 0.00–0.00 |
| 22 | 5 June 2016 | 50 | 0–17 | 0.00–25.27 |
| 23 | 12 June 2016 | 56 | 0–11 | 0.00–15.46 |
| 24 | 19 June 2016 | 55 | 0–13 | 0.00–18.41 |
| 25 | 26 June 2016 | 72 | 0–0 | 0.00–0.00 |
| 26 | 3 July 2016 | 74 | 0–0 | 0.00–0.00 |
| 27 | 10 July 2016 | 71 | 0–0 | 0.00–0.00 |
| 28 | 17 July 2016 | 61 | 0–11 | 0.00–14.58 |
| 29 | 24 July 2016 | 63 | 0–10 | 0.00–13.08 |
| 30 | 31 July 2016 | 69 | 0–4 | 0.00–5.43 |
| 31 | 7 August 2016 | 68 | 0–7 | 0.00–8.31 |
| 32 | 14 August 2016 | 84 | 0–0 | 0.00–0.00 |
| 33 | 21 August 2016 | 72 | 0–3 | 0.00–3.94 |
| 34 | 28 August 2016 | 74 | 0–3 | 0.00–2.83 |
| 35 | 4 September 2016 | 73 | 0–3 | 0.00–3.66 |
| 36 | 11 September 2016 | 74 | 0–4 | 0.00–4.71 |
| 37 | 18 September 2016 | 70 | 0–10 | 0.00–12.41 |
| 38 | 25 September 2016 | 63 | 0–17 | 0.00–21.03 |
| 39 | 2 October 2016 | 87 | 0–0 | 0.00–0.00 |
| 40 | 9 October 2016 | 77 | 0–9 | 0.00–9.56 |
| 41 | 16 October 2016 | 73 | 0–12 | 0.00–13.81 |
| 42 | 23 October 2016 | 94 | 0–0 | 0.00–0.00 |
| 43 | 30 October 2016 | 90 | 0–0 | 0.00–0.00 |
| 44 | 6 November 2016 | 91 | 0–0 | 0.00–0.00 |
| 45 | 13 November 2016 | 77 | 0–13 | 0.00–13.75 |
| 46 | 20 November 2016 | 80 | 0–13 | 0.00–13.36 |
| 47 | 27 November 2016 | 71 | 1–22 | 1.39–23.65 |
| 48 | 4 December 2016 | 97 | 0–0 | 0.00–0.00 |
| 49 | 11 December 2016 | 80 | 0–14 | 0.00–14.08 |
| 50 | 18 December 2016 | 103 | 0–0 | 0.00–0.00 |
| 51 | 25 December 2016 | 80 | 0–9 | 0.00–9.24 |
| 52 | 1 January 2017 | 111 | 0–0 | 0.00–0.00 |
| 2017 |  |  |  |  |
| Week | Week ending date | Observed deaths | Exiguous deaths | Percent deficit |
| 1 | 8 January 2017 | 64 | 0–16 | 0.00–19.58 |
| 2 | 15 January 2017 | 73 | 0–2 | 0.00–1.85 |
| 3 | 22 January 2017 | 62 | 0–9 | 0.00–11.48 |
| 4 | 29 January 2017 | 63 | 0–7 | 0.00–9.49 |
| 5 | 5 February 2017 | 60 | 0–10 | 0.00–13.16 |
| 6 | 12 February 2017 | 56 | 0–13 | 0.00–18.52 |
| 7 | 19 February 2017 | 82 | 0–0 | 0.00–0.00 |
| 8 | 26 February 2017 | 67 | 0–1 | 0.00–1.44 |
| 9 | 5 March 2017 | 80 | 0–0 | 0.00–0.00 |
| 10 | 12 March 2017 | 70 | 0–0 | 0.00–0.00 |
| 11 | 19 March 2017 | 66 | 0–2 | 0.00–2.61 |
| 12 | 26 March 2017 | 62 | 0–6 | 0.00–8.14 |
| 13 | 2 April 2017 | 78 | 0–0 | 0.00–0.00 |
| 14 | 9 April 2017 | 54 | 0–14 | 0.00–20.14 |
| 15 | 16 April 2017 | 60 | 0–8 | 0.00–10.96 |
| 16 | 23 April 2017 | 60 | 0–8 | 0.00–11.33 |
| 17 | 30 April 2017 | 54 | 0–14 | 0.00–19.46 |
| 18 | 7 May 2017 | 63 | 0–4 | 0.00–5.79 |
| 19 | 14 May 2017 | 54 | 0–12 | 0.00–17.89 |
| 20 | 21 May 2017 | 73 | 0–0 | 0.00–0.00 |
| 21 | 28 May 2017 | 60 | 0–4 | 0.00–5.63 |
| 22 | 4 June 2017 | 66 | 0–0 | 0.00–0.00 |
| 23 | 11 June 2017 | 59 | 0–4 | 0.00–5.18 |
| 24 | 18 June 2017 | 57 | 0–6 | 0.00–8.80 |
| 25 | 25 June 2017 | 74 | 0–0 | 0.00–0.00 |
| 26 | 2 July 2017 | 69 | 0–0 | 0.00–0.00 |
| 27 | 9 July 2017 | 63 | 0–2 | 0.00–1.87 |
| 28 | 16 July 2017 | 72 | 0–0 | 0.00–0.00 |
| 29 | 23 July 2017 | 70 | 0–0 | 0.00–0.00 |
| 30 | 30 July 2017 | 76 | 0–0 | 0.00–0.00 |
| 31 | 6 August 2017 | 88 | 0–0 | 0.00–0.00 |
| 32 | 13 August 2017 | 69 | 0–0 | 0.00–0.00 |
| 33 | 20 August 2017 | 52 | 0–16 | 0.00–23.24 |
| 34 | 27 August 2017 | 77 | 0–0 | 0.00–0.00 |
| 35 | 3 September 2017 | 82 | 0–0 | 0.00–0.00 |
| 36 | 10 September 2017 | 65 | 0–8 | 0.00–10.59 |
| 37 | 17 September 2017 | 67 | 0–7 | 0.00–8.58 |
| 38 | 24 September 2017 | 71 | 0–2 | 0.00–2.48 |
| 39 | 1 October 2017 | 64 | 0–12 | 0.00–15.42 |
| 40 | 8 October 2017 | 63 | 0–16 | 0.00–19.51 |
| 41 | 15 October 2017 | 73 | 0–6 | 0.00–6.70 |
| 42 | 22 October 2017 | 87 | 0–0 | 0.00–0.00 |
| 43 | 29 October 2017 | 78 | 0–2 | 0.00–1.58 |
| 44 | 5 November 2017 | 97 | 0–0 | 0.00–0.00 |
| 45 | 12 November 2017 | 78 | 0–5 | 0.00–5.86 |
| 46 | 19 November 2017 | 81 | 0–4 | 0.00–4.50 |
| 47 | 26 November 2017 | 102 | 0–0 | 0.00–0.00 |
| 48 | 3 December 2017 | 79 | 0–8 | 0.00–9.06 |
| 49 | 10 December 2017 | 91 | 0–0 | 0.00–0.00 |
| 50 | 17 December 2017 | 73 | 0–11 | 0.00–13.02 |
| 51 | 24 December 2017 | 87 | 0–0 | 0.00–0.00 |
| 52 | 31 December 2017 | 98 | 0–0 | 0.00–0.00 |
| 2018 |  |  |  |  |
| Week | Week ending date | Observed deaths | Exiguous deaths | Percent deficit |
| 1 | 7 January 2018 | 60 | 0–14 | 0.00–18.57 |
| 2 | 14 January 2018 | 66 | 0–3 | 0.00–3.57 |
| 3 | 21 January 2018 | 96 | 0–0 | 0.00–0.00 |
| 4 | 28 January 2018 | 72 | 0–0 | 0.00–0.00 |
| 5 | 4 February 2018 | 64 | 0–2 | 0.00–2.13 |
| 6 | 11 February 2018 | 53 | 0–13 | 0.00–18.65 |
| 7 | 18 February 2018 | 61 | 0–4 | 0.00–5.63 |
| 8 | 25 February 2018 | 67 | 0–0 | 0.00–0.00 |
| 9 | 4 March 2018 | 65 | 0–0 | 0.00–0.00 |
| 10 | 11 March 2018 | 61 | 0–4 | 0.00–5.92 |
| 11 | 18 March 2018 | 54 | 0–11 | 0.00–15.92 |
| 12 | 25 March 2018 | 63 | 0–2 | 0.00–1.82 |
| 13 | 1 April 2018 | 71 | 0–0 | 0.00–0.00 |
| 14 | 8 April 2018 | 61 | 0–3 | 0.00–4.60 |
| 15 | 15 April 2018 | 59 | 0–5 | 0.00–7.12 |
| 16 | 22 April 2018 | 64 | 0–0 | 0.00–0.00 |
| 17 | 29 April 2018 | 67 | 0–0 | 0.00–0.00 |
| 18 | 6 May 2018 | 63 | 0–1 | 0.00–0.29 |
| 19 | 13 May 2018 | 60 | 0–3 | 0.00–3.94 |
| 20 | 20 May 2018 | 53 | 0–9 | 0.00–14.15 |
| 21 | 27 May 2018 | 55 | 0–7 | 0.00–10.05 |
| 22 | 3 June 2018 | 60 | 0–1 | 0.00–0.75 |
| 23 | 10 June 2018 | 48 | 0–13 | 0.00–20.72 |
| 24 | 17 June 2018 | 52 | 0–10 | 0.00–14.86 |
| 25 | 24 June 2018 | 58 | 0–3 | 0.00–4.83 |
| 26 | 1 July 2018 | 53 | 0–10 | 0.00–14.99 |
| 27 | 8 July 2018 | 55 | 0–8 | 0.00–12.44 |
| 28 | 15 July 2018 | 64 | 0–1 | 0.00–0.23 |
| 29 | 22 July 2018 | 66 | 0–0 | 0.00–0.00 |
| 30 | 29 July 2018 | 68 | 0–0 | 0.00–0.00 |
| 31 | 5 August 2018 | 66 | 0–1 | 0.00–0.05 |
| 32 | 12 August 2018 | 71 | 0–0 | 0.00–0.00 |
| 33 | 19 August 2018 | 66 | 0–0 | 0.00–0.00 |
| 34 | 26 August 2018 | 50 | 1–18 | 1.96–25.96 |
| 35 | 2 September 2018 | 79 | 0–0 | 0.00–0.00 |
| 36 | 9 September 2018 | 59 | 0–10 | 0.00–13.42 |
| 37 | 16 September 2018 | 67 | 0–3 | 0.00–3.18 |
| 38 | 23 September 2018 | 88 | 0–0 | 0.00–0.00 |
| 39 | 30 September 2018 | 57 | 0–15 | 0.00–19.75 |
| 40 | 7 October 2018 | 70 | 0–3 | 0.00–4.10 |
| 41 | 14 October 2018 | 76 | 0–0 | 0.00–0.00 |
| 42 | 21 October 2018 | 88 | 0–0 | 0.00–0.00 |
| 43 | 28 October 2018 | 72 | 0–4 | 0.00–5.19 |
| 44 | 4 November 2018 | 84 | 0–0 | 0.00–0.00 |
| 45 | 11 November 2018 | 84 | 0–0 | 0.00–0.00 |
| 46 | 18 November 2018 | 68 | 0–14 | 0.00–16.42 |
| 47 | 25 November 2018 | 57 | 6–25 | 9.52–30.03 |
| 48 | 2 December 2018 | 88 | 0–0 | 0.00–0.00 |
| 49 | 9 December 2018 | 83 | 0–0 | 0.00–0.00 |
| 50 | 16 December 2018 | 90 | 0–0 | 0.00–0.00 |
| 51 | 23 December 2018 | 89 | 0–0 | 0.00–0.00 |
| 52 | 30 December 2018 | 119 | 0–0 | 0.00–0.00 |
| 2019 |  |  |  |  |
| Week | Week ending date | Observed deaths | Exiguous deaths | Percent deficit |
| 1 | 6 January 2019 | 48 | 7–26 | 12.73–34.27 |
| 2 | 13 January 2019 | 61 | 0–8 | 0.00–10.91 |
| 3 | 20 January 2019 | 75 | 0–0 | 0.00–0.00 |
| 4 | 27 January 2019 | 55 | 0–8 | 0.00–11.80 |
| 5 | 3 February 2019 | 55 | 0–7 | 0.00–10.96 |
| 6 | 10 February 2019 | 49 | 0–12 | 0.00–18.90 |
| 7 | 17 February 2019 | 50 | 0–12 | 0.00–18.11 |
| 8 | 24 February 2019 | 55 | 0–6 | 0.00–8.87 |
| 9 | 3 March 2019 | 57 | 0–4 | 0.00–5.26 |
| 10 | 10 March 2019 | 55 | 0–7 | 0.00–10.25 |
| 11 | 17 March 2019 | 62 | 0–0 | 0.00–0.00 |
| 12 | 24 March 2019 | 68 | 0–0 | 0.00–0.00 |
| 13 | 31 March 2019 | 53 | 0–8 | 0.00–12.92 |
| 14 | 7 April 2019 | 67 | 0–0 | 0.00–0.00 |
| 15 | 14 April 2019 | 52 | 0–9 | 0.00–13.93 |
| 16 | 21 April 2019 | 72 | 0–0 | 0.00–0.00 |
| 17 | 28 April 2019 | 65 | 0–0 | 0.00–0.00 |
| 18 | 5 May 2019 | 43 | 2–17 | 4.44–27.91 |
| 19 | 12 May 2019 | 54 | 0–5 | 0.00–7.76 |
| 20 | 19 May 2019 | 32 | 10–26 | 23.81–44.39 |
| 21 | 26 May 2019 | 58 | 0–0 | 0.00–0.00 |
| 22 | 2 June 2019 | 45 | 0–12 | 0.00–19.71 |
| 23 | 9 June 2019 | 54 | 0–3 | 0.00–3.77 |
| 24 | 16 June 2019 | 50 | 0–7 | 0.00–11.22 |
| 25 | 23 June 2019 | 43 | 0–14 | 0.00–23.98 |
| 26 | 30 June 2019 | 47 | 0–11 | 0.00–18.94 |
| 27 | 7 July 2019 | 54 | 0–6 | 0.00–9.65 |
| 28 | 14 July 2019 | 42 | 3–20 | 6.67–31.40 |
| 29 | 21 July 2019 | 48 | 0–14 | 0.00–21.89 |
| 30 | 28 July 2019 | 56 | 0–6 | 0.00–8.59 |
| 31 | 4 August 2019 | 55 | 0–7 | 0.00–10.84 |
| 32 | 11 August 2019 | 69 | 0–0 | 0.00–0.00 |
| 33 | 18 August 2019 | 49 | 0–13 | 0.00–20.91 |
| 34 | 25 August 2019 | 79 | 0–0 | 0.00–0.00 |
| 35 | 1 September 2019 | 64 | 0–0 | 0.00–0.00 |
| 36 | 8 September 2019 | 70 | 0–0 | 0.00–0.00 |
| 37 | 15 September 2019 | 73 | 0–0 | 0.00–0.00 |
| 38 | 22 September 2019 | 69 | 0–0 | 0.00–0.00 |
| 39 | 29 September 2019 | 62 | 0–5 | 0.00–6.54 |
| 40 | 6 October 2019 | 54 | 0–15 | 0.00–20.79 |
| 41 | 13 October 2019 | 62 | 0–8 | 0.00–10.41 |
| 42 | 20 October 2019 | 70 | 0–0 | 0.00–0.00 |
| 43 | 27 October 2019 | 82 | 0–0 | 0.00–0.00 |
| 44 | 3 November 2019 | 99 | 0–0 | 0.00–0.00 |
| 45 | 10 November 2019 | 70 | 0–4 | 0.00–4.94 |
| 46 | 17 November 2019 | 73 | 0–1 | 0.00–0.42 |
| 47 | 24 November 2019 | 79 | 0–0 | 0.00–0.00 |
| 48 | 1 December 2019 | 70 | 0–5 | 0.00–5.91 |
| 49 | 8 December 2019 | 75 | 0–1 | 0.00–0.21 |
| 50 | 15 December 2019 | 78 | 0–0 | 0.00–0.00 |
| 51 | 22 December 2019 | 95 | 0–0 | 0.00–0.00 |
| 52 | 29 December 2019 | 79 | 0–0 | 0.00–0.00 |

Percent deficit during the COVID-19 pandemic were defined as the number of deficit deaths divided by the threshold. Weeks with observed deaths from road injuries falling the 95% lower bound were highlighted in gray.

**Supplementary table 2: Weekly number of observed and deficit deaths and percent deficit from road injuries in seven prefectures where a state of emergency was first declared on 7 April 2020, from January 2016 to 2019.**

| 2016 |  |  |  |  |
| --- | --- | --- | --- | --- |
| Week | Week ending date | Observed deaths | Exiguous deaths | Percent deficit |
| 1 | 2016 January 10 | 24 | 0–1 | 0.00–0.79 |
| 2 | 17 January 2016 | 27 | 0–0 | 0.00–0.00 |
| 3 | 24 January 2016 | 15 | 0–7 | 0.00–28.76 |
| 4 | 31 January 2016 | 24 | 0–0 | 0.00–0.00 |
| 5 | 7 February 2016 | 17 | 0–4 | 0.00–17.56 |
| 6 | 14 February 2016 | 16 | 0–5 | 0.00–20.79 |
| 7 | 21 February 2016 | 20 | 0–1 | 0.00–1.64 |
| 8 | 28 February 2016 | 10 | 2–11 | 16.67–51.23 |
| 9 | 6 March 2016 | 16 | 0–5 | 0.00–21.74 |
| 10 | 13 March 2016 | 21 | 0–0 | 0.00–0.00 |
| 11 | 20 March 2016 | 17 | 0–4 | 0.00–17.15 |
| 12 | 27 March 2016 | 21 | 0–0 | 0.00–0.00 |
| 13 | 3 April 2016 | 22 | 0–0 | 0.00–0.00 |
| 14 | 10 April 2016 | 21 | 0–0 | 0.00–0.00 |
| 15 | 17 April 2016 | 24 | 0–0 | 0.00–0.00 |
| 16 | 24 April 2016 | 22 | 0–0 | 0.00–0.00 |
| 17 | 1 May 2016 | 18 | 0–2 | 0.00–8.82 |
| 18 | 8 May 2016 | 20 | 0–0 | 0.00–0.00 |
| 19 | 15 May 2016 | 17 | 0–4 | 0.00–16.84 |
| 20 | 22 May 2016 | 11 | 0–9 | 0.00–44.21 |
| 21 | 29 May 2016 | 21 | 0–0 | 0.00–0.00 |
| 22 | 5 June 2016 | 13 | 0–8 | 0.00–35.80 |
| 23 | 12 June 2016 | 18 | 0–3 | 0.00–11.99 |
| 24 | 19 June 2016 | 17 | 0–4 | 0.00–17.47 |
| 25 | 26 June 2016 | 18 | 0–3 | 0.00–12.21 |
| 26 | 3 July 2016 | 15 | 0–5 | 0.00–24.25 |
| 27 | 10 July 2016 | 22 | 0–0 | 0.00–0.00 |
| 28 | 17 July 2016 | 16 | 0–5 | 0.00–21.18 |
| 29 | 24 July 2016 | 16 | 0–5 | 0.00–21.32 |
| 30 | 31 July 2016 | 23 | 0–0 | 0.00–0.00 |
| 31 | 7 August 2016 | 25 | 0–0 | 0.00–0.00 |
| 32 | 14 August 2016 | 20 | 0–2 | 0.00–5.87 |
| 33 | 21 August 2016 | 18 | 0–4 | 0.00–16.62 |
| 34 | 28 August 2016 | 23 | 0–0 | 0.00–0.00 |
| 35 | 4 September 2016 | 26 | 0–0 | 0.00–0.00 |
| 36 | 11 September 2016 | 15 | 0–8 | 0.00–34.26 |
| 37 | 18 September 2016 | 15 | 0–10 | 0.00–38.01 |
| 38 | 25 September 2016 | 24 | 0–0 | 0.00–0.00 |
| 39 | 2 October 2016 | 25 | 0–0 | 0.00–0.00 |
| 40 | 9 October 2016 | 28 | 0–0 | 0.00–0.00 |
| 41 | 16 October 2016 | 15 | 0–10 | 0.00–38.89 |
| 42 | 23 October 2016 | 29 | 0–0 | 0.00–0.00 |
| 43 | 30 October 2016 | 23 | 0–3 | 0.00–8.79 |
| 44 | 6 November 2016 | 28 | 0–0 | 0.00–0.00 |
| 45 | 13 November 2016 | 18 | 0–9 | 0.00–31.34 |
| 46 | 20 November 2016 | 22 | 0–5 | 0.00–17.60 |
| 47 | 27 November 2016 | 21 | 0–7 | 0.00–24.61 |
| 48 | 4 December 2016 | 23 | 0–7 | 0.00–22.48 |
| 49 | 11 December 2016 | 26 | 0–3 | 0.00–9.92 |
| 50 | 18 December 2016 | 29 | 0–0 | 0.00–0.00 |
| 51 | 25 December 2016 | 26 | 0–1 | 0.00–2.39 |
| 52 | 1 January 2017 | 39 | 0–0 | 0.00–0.00 |
| 2017 |  |  |  |  |
| Week | Week ending date | Observed deaths | Exiguous deaths | Percent deficit |
| 1 | 8 January 2017 | 16 | 0–8 | 0.00–31.65 |
| 2 | 15 January 2017 | 25 | 0–0 | 0.00–0.00 |
| 3 | 22 January 2017 | 18 | 0–3 | 0.00–10.64 |
| 4 | 29 January 2017 | 17 | 0–3 | 0.00–14.68 |
| 5 | 5 February 2017 | 18 | 0–2 | 0.00–7.26 |
| 6 | 12 February 2017 | 25 | 0–0 | 0.00–0.00 |
| 7 | 19 February 2017 | 29 | 0–0 | 0.00–0.00 |
| 8 | 26 February 2017 | 18 | 0–1 | 0.00–4.59 |
| 9 | 5 March 2017 | 22 | 0–0 | 0.00–0.00 |
| 10 | 12 March 2017 | 27 | 0–0 | 0.00–0.00 |
| 11 | 19 March 2017 | 17 | 0–3 | 0.00–12.40 |
| 12 | 26 March 2017 | 22 | 0–0 | 0.00–0.00 |
| 13 | 2 April 2017 | 23 | 0–0 | 0.00–0.00 |
| 14 | 9 April 2017 | 13 | 0–8 | 0.00–36.16 |
| 15 | 16 April 2017 | 13 | 0–7 | 0.00–34.69 |
| 16 | 23 April 2017 | 20 | 0–0 | 0.00–0.00 |
| 17 | 30 April 2017 | 10 | 1–10 | 9.09–47.63 |
| 18 | 7 May 2017 | 18 | 0–2 | 0.00–5.89 |
| 19 | 14 May 2017 | 13 | 0–7 | 0.00–32.20 |
| 20 | 21 May 2017 | 13 | 0–6 | 0.00–28.71 |
| 21 | 28 May 2017 | 20 | 0–0 | 0.00–0.00 |
| 22 | 4 June 2017 | 19 | 0–0 | 0.00–0.00 |
| 23 | 11 June 2017 | 17 | 0–2 | 0.00–7.26 |
| 24 | 18 June 2017 | 20 | 0–0 | 0.00–0.00 |
| 25 | 25 June 2017 | 23 | 0–0 | 0.00–0.00 |
| 26 | 2 July 2017 | 16 | 0–2 | 0.00–8.54 |
| 27 | 9 July 2017 | 20 | 0–0 | 0.00–0.00 |
| 28 | 16 July 2017 | 18 | 0–0 | 0.00–0.00 |
| 29 | 23 July 2017 | 16 | 0–3 | 0.00–11.96 |
| 30 | 30 July 2017 | 30 | 0–0 | 0.00–0.00 |
| 31 | 6 August 2017 | 26 | 0–0 | 0.00–0.00 |
| 32 | 13 August 2017 | 15 | 0–5 | 0.00–21.53 |
| 33 | 20 August 2017 | 10 | 1–10 | 9.09–48.33 |
| 34 | 27 August 2017 | 23 | 0–0 | 0.00–0.00 |
| 35 | 3 September 2017 | 24 | 0–0 | 0.00–0.00 |
| 36 | 10 September 2017 | 21 | 0–1 | 0.00–1.85 |
| 37 | 17 September 2017 | 20 | 0–3 | 0.00–11.95 |
| 38 | 24 September 2017 | 21 | 0–1 | 0.00–3.16 |
| 39 | 1 October 2017 | 18 | 0–5 | 0.00–19.05 |
| 40 | 8 October 2017 | 20 | 0–4 | 0.00–14.12 |
| 41 | 15 October 2017 | 24 | 0–0 | 0.00–0.00 |
| 42 | 22 October 2017 | 27 | 0–0 | 0.00–0.00 |
| 43 | 29 October 2017 | 17 | 0–7 | 0.00–26.84 |
| 44 | 5 November 2017 | 43 | 0–0 | 0.00–0.00 |
| 45 | 12 November 2017 | 22 | 0–3 | 0.00–10.75 |
| 46 | 19 November 2017 | 25 | 0–0 | 0.00–0.00 |
| 47 | 26 November 2017 | 31 | 0–0 | 0.00–0.00 |
| 48 | 3 December 2017 | 25 | 0–2 | 0.00–6.72 |
| 49 | 10 December 2017 | 30 | 0–0 | 0.00–0.00 |
| 50 | 17 December 2017 | 22 | 0–4 | 0.00–15.23 |
| 51 | 24 December 2017 | 27 | 0–0 | 0.00–0.00 |
| 52 | 31 December 2017 | 26 | 0–0 | 0.00–0.00 |
| 2018 |  |  |  |  |
| Week | Week ending date | Observed deaths | Exiguous deaths | Percent deficit |
| 1 | 7 January 2018 | 19 | 0–4 | 0.00–14.89 |
| 2 | 14 January 2018 | 20 | 0–1 | 0.00–1.53 |
| 3 | 21 January 2018 | 37 | 0–0 | 0.00–0.00 |
| 4 | 28 January 2018 | 25 | 0–0 | 0.00–0.00 |
| 5 | 4 February 2018 | 22 | 0–0 | 0.00–0.00 |
| 6 | 11 February 2018 | 22 | 0–0 | 0.00–0.00 |
| 7 | 18 February 2018 | 28 | 0–0 | 0.00–0.00 |
| 8 | 25 February 2018 | 20 | 0–0 | 0.00–0.00 |
| 9 | 4 March 2018 | 21 | 0–0 | 0.00–0.00 |
| 10 | 11 March 2018 | 20 | 0–0 | 0.00–0.00 |
| 11 | 18 March 2018 | 18 | 0–1 | 0.00–4.61 |
| 12 | 25 March 2018 | 12 | 0–8 | 0.00–36.89 |
| 13 | 1 April 2018 | 15 | 0–5 | 0.00–22.72 |
| 14 | 8 April 2018 | 19 | 0–1 | 0.00–0.17 |
| 15 | 15 April 2018 | 14 | 0–5 | 0.00–24.50 |
| 16 | 22 April 2018 | 25 | 0–0 | 0.00–0.00 |
| 17 | 29 April 2018 | 20 | 0–0 | 0.00–0.00 |
| 18 | 6 May 2018 | 20 | 0–0 | 0.00–0.00 |
| 19 | 13 May 2018 | 24 | 0–0 | 0.00–0.00 |
| 20 | 20 May 2018 | 11 | 0–7 | 0.00–36.30 |
| 21 | 27 May 2018 | 11 | 0–7 | 0.00–36.39 |
| 22 | 3 June 2018 | 22 | 0–0 | 0.00–0.00 |
| 23 | 10 June 2018 | 15 | 0–3 | 0.00–14.21 |
| 24 | 17 June 2018 | 14 | 0–4 | 0.00–21.48 |
| 25 | 24 June 2018 | 13 | 0–5 | 0.00–25.62 |
| 26 | 1 July 2018 | 26 | 0–0 | 0.00–0.00 |
| 27 | 8 July 2018 | 16 | 0–2 | 0.00–9.36 |
| 28 | 15 July 2018 | 19 | 0–0 | 0.00–0.00 |
| 29 | 22 July 2018 | 15 | 0–3 | 0.00–15.23 |
| 30 | 29 July 2018 | 17 | 0–1 | 0.00–3.04 |
| 31 | 5 August 2018 | 24 | 0–0 | 0.00–0.00 |
| 32 | 12 August 2018 | 23 | 0–0 | 0.00–0.00 |
| 33 | 19 August 2018 | 17 | 0–3 | 0.00–10.80 |
| 34 | 26 August 2018 | 16 | 0–4 | 0.00–16.84 |
| 35 | 2 September 2018 | 23 | 0–0 | 0.00–0.00 |
| 36 | 9 September 2018 | 22 | 0–0 | 0.00–0.00 |
| 37 | 16 September 2018 | 17 | 0–5 | 0.00–20.76 |
| 38 | 23 September 2018 | 25 | 0–0 | 0.00–0.00 |
| 39 | 30 September 2018 | 17 | 0–5 | 0.00–19.46 |
| 40 | 7 October 2018 | 22 | 0–0 | 0.00–0.00 |
| 41 | 14 October 2018 | 23 | 0–0 | 0.00–0.00 |
| 42 | 21 October 2018 | 29 | 0–0 | 0.00–0.00 |
| 43 | 28 October 2018 | 25 | 0–0 | 0.00–0.00 |
| 44 | 4 November 2018 | 32 | 0–0 | 0.00–0.00 |
| 45 | 11 November 2018 | 21 | 0–4 | 0.00–13.58 |
| 46 | 18 November 2018 | 21 | 0–4 | 0.00–15.88 |
| 47 | 25 November 2018 | 23 | 0–3 | 0.00–11.53 |
| 48 | 2 December 2018 | 21 | 0–5 | 0.00–18.87 |
| 49 | 9 December 2018 | 26 | 0–1 | 0.00–1.74 |
| 50 | 16 December 2018 | 24 | 0–2 | 0.00–6.57 |
| 51 | 23 December 2018 | 32 | 0–0 | 0.00–0.00 |
| 52 | 30 December 2018 | 38 | 0–0 | 0.00–0.00 |
| 2019 |  |  |  |  |
| Week | Week ending date | Observed deaths | Exiguous deaths | Percent deficit |
| 1 | 6 January 2019 | 12 | 2–12 | 14.29–48.47 |
| 2 | 13 January 2019 | 20 | 0–2 | 0.00–4.88 |
| 3 | 20 January 2019 | 25 | 0–0 | 0.00–0.00 |
| 4 | 27 January 2019 | 20 | 0–0 | 0.00–0.00 |
| 5 | 3 February 2019 | 12 | 0–7 | 0.00–36.79 |
| 6 | 10 February 2019 | 15 | 0–4 | 0.00–19.19 |
| 7 | 17 February 2019 | 17 | 0–3 | 0.00–11.49 |
| 8 | 24 February 2019 | 12 | 0–7 | 0.00–36.25 |
| 9 | 3 March 2019 | 11 | 0–8 | 0.00–40.89 |
| 10 | 10 March 2019 | 21 | 0–0 | 0.00–0.00 |
| 11 | 17 March 2019 | 27 | 0–0 | 0.00–0.00 |
| 12 | 24 March 2019 | 15 | 0–4 | 0.00–17.01 |
| 13 | 31 March 2019 | 15 | 0–4 | 0.00–17.66 |
| 14 | 7 April 2019 | 16 | 0–2 | 0.00–9.58 |
| 15 | 14 April 2019 | 12 | 0–6 | 0.00–31.17 |
| 16 | 21 April 2019 | 24 | 0–0 | 0.00–0.00 |
| 17 | 28 April 2019 | 22 | 0–0 | 0.00–0.00 |
| 18 | 5 May 2019 | 8 | 1–9 | 11.11–52.29 |
| 19 | 12 May 2019 | 12 | 0–6 | 0.00–29.89 |
| 20 | 19 May 2019 | 10 | 0–7 | 0.00–38.62 |
| 21 | 26 May 2019 | 18 | 0–0 | 0.00–0.00 |
| 22 | 2 June 2019 | 12 | 0–5 | 0.00–26.32 |
| 23 | 9 June 2019 | 14 | 0–3 | 0.00–12.63 |
| 24 | 16 June 2019 | 9 | 0–8 | 0.00–45.46 |
| 25 | 23 June 2019 | 8 | 1–9 | 11.11–51.37 |
| 26 | 30 June 2019 | 14 | 0–3 | 0.00–13.75 |
| 27 | 7 July 2019 | 19 | 0–0 | 0.00–0.00 |
| 28 | 14 July 2019 | 15 | 0–3 | 0.00–12.41 |
| 29 | 21 July 2019 | 10 | 0–8 | 0.00–42.55 |
| 30 | 28 July 2019 | 17 | 0–1 | 0.00–0.63 |
| 31 | 4 August 2019 | 17 | 0–1 | 0.00–3.39 |
| 32 | 11 August 2019 | 18 | 0–1 | 0.00–1.19 |
| 33 | 18 August 2019 | 15 | 0–4 | 0.00–17.31 |
| 34 | 25 August 2019 | 23 | 0–0 | 0.00–0.00 |
| 35 | 1 September 2019 | 22 | 0–0 | 0.00–0.00 |
| 36 | 8 September 2019 | 18 | 0–1 | 0.00–0.57 |
| 37 | 15 September 2019 | 20 | 0–0 | 0.00–0.00 |
| 38 | 22 September 2019 | 23 | 0–0 | 0.00–0.00 |
| 39 | 29 September 2019 | 16 | 0–4 | 0.00–17.70 |
| 40 | 6 October 2019 | 17 | 0–3 | 0.00–14.97 |
| 41 | 13 October 2019 | 18 | 0–4 | 0.00–15.06 |
| 42 | 20 October 2019 | 17 | 0–5 | 0.00–19.52 |
| 43 | 27 October 2019 | 24 | 0–0 | 0.00–0.00 |
| 44 | 3 November 2019 | 18 | 0–4 | 0.00–16.32 |
| 45 | 10 November 2019 | 16 | 0–6 | 0.00–27.08 |
| 46 | 17 November 2019 | 30 | 0–0 | 0.00–0.00 |
| 47 | 24 November 2019 | 28 | 0–0 | 0.00–0.00 |
| 48 | 1 December 2019 | 16 | 0–8 | 0.00–31.09 |
| 49 | 8 December 2019 | 27 | 0–0 | 0.00–0.00 |
| 50 | 15 December 2019 | 33 | 0–0 | 0.00–0.00 |
| 51 | 22 December 2019 | 28 | 0–0 | 0.00–0.00 |
| 52 | 29 December 2019 | 17 | 0–7 | 0.00–26.65 |

Percent deficit during the COVID-19 pandemic were defined as the number of deficit deaths divided by the threshold. Weeks with observed deaths from road injuries falling the 95% lower bound were highlighted in gray.
